# Physical-Chemical Evaluation of Active Food Packaging Material Based on Thermoplastic Starch Loaded with Grape cane Extract

**DOI:** 10.3390/molecules25061306

**Published:** 2020-03-13

**Authors:** Edaena Pamela Díaz-Galindo, Aleksandra Nesic, Gustavo Cabrera-Barjas, Claudia Mardones, Dietrich von Baer, Silvia Bautista-Baños, Octavio Dublan Garcia

**Affiliations:** 1 Facultad de Química, Universidad Autónoma del Estado de México, Km 115 Carr. Toluca-Ixtlahuaca. El Cerrillo Piedras Blancas, Toluca 50100, Mexico; pam.dg12@hotmail.com (E.P.D.-G.); odublang@uaemex.mx (O.D.G.); 2 Unidad de Desarrollo Tecnológico (UDT), Universidad de Concepción, Avda. Cordillera No. 2634, Parque Industrial Coronel, Coronel 4191996, Chile; a.nesic@udt.cl; 3 Vinca Institute for Nuclear Sciences, University of Belgrade, Mike Petrovica-Alasa 12-14, 11000 Belgrade, Serbia; 4 Departamento de Análisis Instrumental, Universidad de Concepción, Barrio Universitario s/n, Concepción P.O-Box 160-C, Concepción 4070386, Chile; cmardones@udec.cl (C.M.); dvonbaer@udec.cl (D.v.B.); 5 Centro de Desarrollo de Productos Bióticos (CEPROBI), Instituto Politécnico Nacional. Carretera Yautepec-Jojutla, Km. 6, calle CEPROBI No. 8, Col. San Isidro, Yautepec, Morelos 62731, Mexico; sbautis@ipn.mx

**Keywords:** thermoplastic starch, grape cane extract, active food packaging, stilbenoids, resveratrol

## Abstract

The aim of this paper is to evaluate the physicochemical and microbiological properties of active thermoplastic starch-based materials. The extract obtained from grape cane waste was used as a source of stilbene bioactive components to enhance the functional properties of thermoplastic starch (TPS). The biomaterials were prepared by the compression molding technique and subjected to mechanical, thermal, antioxidant, and microbiological tests. The results showed that the addition of grape cane extract up to 15 wt% (TPS/WE15) did not significantly influence the thermal stability of obtained biomaterials, whereas mechanical resistance decreased. On the other side, among all tested pathogens, thermoplastic starch based materials showed antifungal activity toward *Botrytis cinerea* and antimicrobial activity toward *Staphylococcus aureus*, suggesting potential application in food packaging as an active biomaterial layer.

## 1. Introduction

Environmental pollution and the management of accumulated waste has become one of the major global problems of contemporary society. World production of plastics grew up to 335 million tons in 2016, where 70% of the total amount of plastic packaging ended up in landfills [1]. Every year, millions of tons of food packaging waste has been generated in landfills, which presents a serious environmental problem. Therefore, the use of biopolymers to produce environmentally sustainable packaging can be a promising solution, as an alternative to plastic packaging. Namely, by use of biopolymers, it can reduce the problem of plastic waste accumulation, as well as a quantity of biomass and agro-industrial waste, from which biopolymers are mostly derived.

In addition to biobased food packaging, there is an increased expansion of active packaging on the market [2]. The active packaging material is designed to release active ingredients into food or absorbs them from food, in order to maintain or improve the quality and shelf life of products. Most famous and commonly used active packaging technologies in the food industry are emitters (antimicrobial agents, antioxidant agents) and absorbers (bind ethylene, moisture, CO_2_ and O_2_) [3]. Active components or absorbers can be used in the form of a pouch/capsule inserted into packaging or can be incorporated into the packaging material itself [4]. Biopolymers have found significant application as active packaging materials, since they can provide high antimicrobial activity, or can improve this functionality by the incorporation of natural active agents into their matrix [5,6,7,8,9,10].

Currently, thermoplastic starch (TPS) is one of the most investigated biopolymers for food packaging application, due to easy processing by commonly used equipment for manufacturing of the plastic materials, such as injection molding, blown film extrusion, compression molding, casting, and extrusion [11,12]. Moreover, starch is a widely abundant biopolymer, since it is obtained from renewable plant resources such as corn, wheat and potato harvests. Generally, TPS is obtained by the disruption of the starch granules and their constitutive crystals into a flowable thermoplastic in the presence of water and plasticizer and under applied high temperature and shear. The main drawbacks that limit the widespread use of TPS packaging are their high sensitivity to moisture and aging within the time, due to starch retrogradation. Hence, TPS materials are available in the food packaging sector only for short life applications, dry food and long shelf life applications that do not require high water vapor/oxygen barriers. The main drawbacks that limit the widespread use of starch-based packaging are their high sensitivity to moisture and aging within the time, due to starch retrogradation. In order to extend the functionality of starch-based materials, different natural fillers and antimicrobial additives have been investigated [13,14,15,16,17,18,19,20,21,22,23,24]. A potential green source of natural additives can be grapevine cane, which represents the agro-industrial waste from viticulture. Grapevine canes are pruned annually and usually disposed into the soil or burned, thus offering no major economic benefits. Recently, it has been found that vine cane waste is rich in bioactive components, mainly trans-resveratrol and trans-ξ-viniferin, that have high antifungal activity toward various pathogens [25,26,27,28]. Hence, the aim of this study was to evaluate the physicochemical properties of biobased materials prepared from corn starch loaded with different content of grapevine cane extract in order to assess their potential use as an active packaging system. Up to date, grape waste, such as seeds [29] and pomace [30,31], have been investigated as a source of polyphenols to upgrade the properties of starch-based materials, but to the best of our knowledge, it is the first time grape cane waste has been used as a filler into a starch matrix.

## 2. Results and Discussion

### 2.1. FTIR Analysis

**Figure 1:**
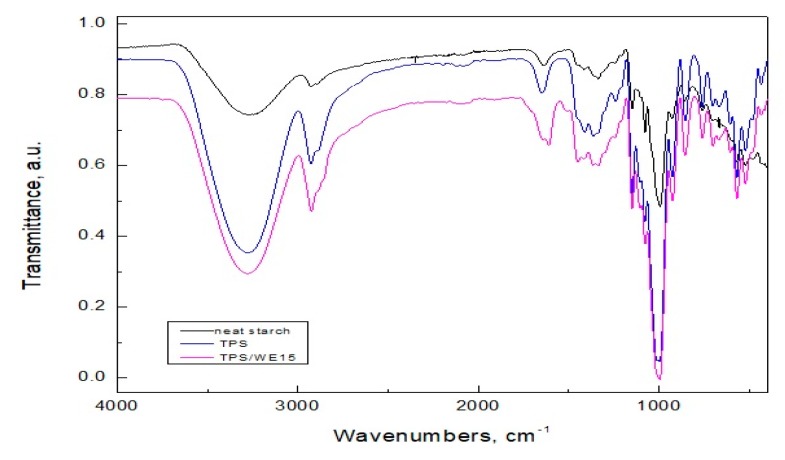
FTIR spectra of starch and starch/grape cane extract samples.

FTIR spectroscopy is a technique that can give insight into starch granules transformation during thermal processing and detects possible interactions between components in material. Hence, in order to confirm the plasticization of starch and to evaluate interactions between grape cane extract and starch, FTIR analysis of neat powder starch, compression-molded starch material (TPS) and thermoplastic starch/grape cane extract material (TPS/WE) was performed and presented in Figure 1. The broad band between 3600 and 3000 cm^−1^ in the spectrum of neat starch is related to stretching vibrations of the OH groups. The band located at 998 cm^−1^ is attributed to the C-O stretching vibrations of the C-O-C group, whereas bands located at 1152 and 1082 cm^−1^ correspond to the C-O stretching vibrations of the C-O-H group. The band at 1645 cm^−1^ is ascribed by δ(O-H) groups from water. It has been reported in the literature that the absorbance band around 1050 cm^−1^ represents the amount of crystalline structure, and the bands around 1020 and 995 cm^−1^ are characteristic of the amorphous starch [32]. Thermal processing of starch causes a shift of bands located around 1020 and 995 cm^−1^ to higher frequencies, whereas the band located around 1050 cm^−1^ completely disappears in the case of the TPS sample. A new peak located at 1105 cm^−1^ has been detected in the spectrum of the S sample, confirming the presence of glycerol [33]. Moreover, the band in the region of 3600 and 3000 cm^−1^ becomes broader, with higher intensity, and this band shifts to higher frequencies after thermal processing of starch, indicating the formation of new hydrogen bonds between water, glycerol, and starch. Regarding the spectra of TPS/WE samples, additional peaks have been detected, i.e., a 1608 cm^−1^ (stretching vibrations of C-C aromatic double group) and 1508 cm^−1^ (in-plane bending vibrations of phenyl C−H bonds) [34,35]. It was reported in the literature that starch could interact with phenolic compounds through the formation of a V-type inclusion complex where the phenolic compound is tightly complexed inside the cavity of amylose helices or through the formation of a complex with much weaker binding mostly through hydrogen bonds [36]. The incorporation of grape cane extract into the starch matrix causes shifts of bands associated with aromatic moieties and (OH) groups, suggesting interactions between starch and bioactive components from the extract via hydrogen bonding and inclusion complexation.

### 2.2. SEM Analysis

**Figure 2:**
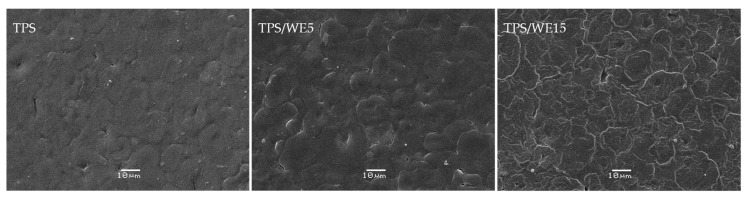
SEM morphology of TPS and TPS/WE materials.

The morphology of thermoplastic starch-based materials is shown in Figure 2. All tested materials demonstrate a rough and dense surface. Moreover, it can be seen that the addition of extract into the starch matrix causes a more dense structure. In fact, a higher concentration of extract leads to its agglomeration in the starch matrix. It is important to note that the compression molding process started the process of losing the structural order in native starch granules and their constitutive crystals. Still, the melt of all crystals is not achieved, which is evidenced by random half-melted crystals on the SEM micrograph (SEM of control TPS). Hence, it was possible to convert starch into thermoplastic starch in a compression molding machine, but full conversion did not occur. In order to obtain a full conversion, the extrusion step before compression molding is required for better homogenization of gelatinized starch with water and glycerol and/or longer time of processing in the compression molding machine. However, both steps can influence the degradation of active components from extracts during the processing of material, giving as a final result material with less bioactive potential. Hence, in this work, compromise with respect to the extract activity has been made, keeping the processing of material as simple as it is possible and with the shortest processing time required to obtain material.

### 2.3. Mechanical Analysis

**Figure 3:**
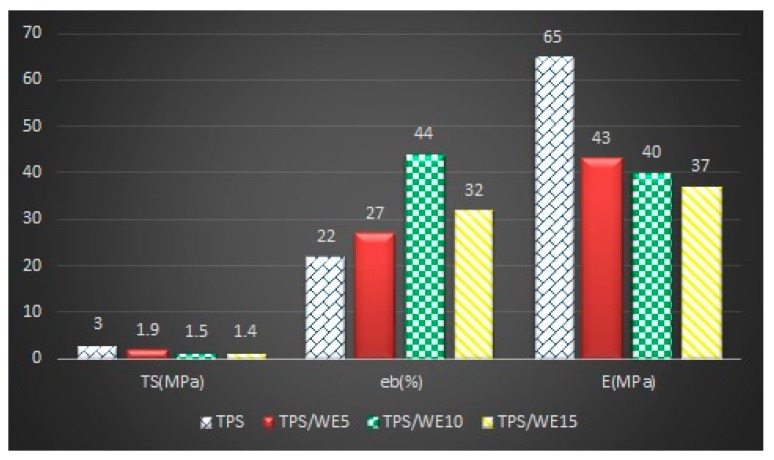
Mechanical parameters obtained for TPS and TPS/WE samples.

Mechanical resistance is a key parameter for food packaging because the package should maintain its integrity during packaging, transport and storage of food products. The mechanical properties of starch materials are presented in Figure 3. The results demonstrate that the addition of grape cane extract up to 15 wt% causes a decrease in tensile strength and Young’s modulus. On the other side, elongation at the break increases for the samples that contain up to the 10 wt% of the extract. Further increase of the extract content in the thermoplastic starch matrix leads to a decrease in elongation at break value. The increase of elongation at break of TPS/WE samples up to specific content can be explained by weakening the intermolecular bonds between the starch chains. Hence, the segmental mobility of starch chains increases, thus leading to improved flexibility and reduced tensile strength and Young’s modulus of TPS/WE5 and TPS/WE10 samples. According to these results, it can be concluded that grape cane extract has an additional plasticizing effect on starch, which has been expected since the extract is rich in polyphenols. The obtained results are in agreement with data from the literature, where the presence of extracts rich in polyphenols (thymol [37], blackberry pulp [38], carvacrol [39], grape pomace waste [30]) increased elongation at break and decreased the tensile strength of starch films. Moreover, Silva et al. showed that the addition of resveratrol into the cellulose matrix led to reduced tensile strength and enhanced elasticity [40]. However, it is important to note that the concentration of extracts in the starch matrix from the above-mentioned literature did not exceed 10 wt%. In the case of the sample TPS/WE15, tensile strength and elongation at the break decrease, probably due to higher agglomeration of grape cane extract particles and their non-homogeneous distribution within the starch matrix.

### 2.4. Thermal Analysis

**Figure 4:**
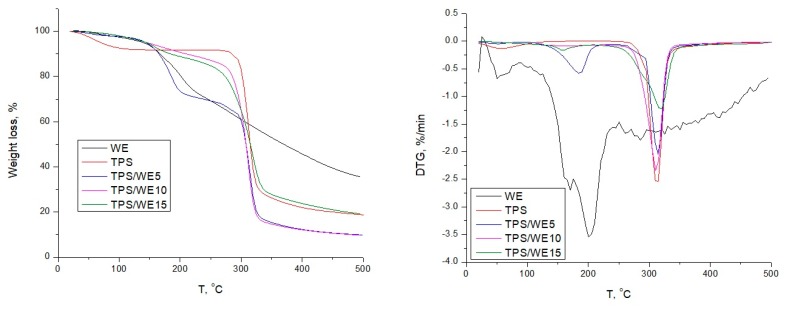
TG and DTG diagrams of TPS and TPS/WE samples.

Thermogravimetric analysis was carried out in order to evaluate the influence of extract on the thermal decomposition of starch-based films. As it can be seen from Figure 4 and Table 1, thermoplastic starch and their composites decompose in three weight loss steps: a) weight loss in the range of 50 °C and 100 °C associated with evaporation of free water, b) weight loss in the range between 100 °C and 180 °C associated with release of bounded water in system, and c) weight loss between 280 °C and 380 °C, associated with degradation of glycerol and starch chains. Grape cane extract shows one degradation step in the range of 120 and 220 °C, related to the decomposition of polyphenols, and also a wide degradation peak in the range of 240–390 °C, which is related to degradation of active components: trans-resveratrol and trans-viniferin [41,42]. The initial degradation temperature (T_onset_) of control thermoplastic starch is detected at 280 °C, whereas the maximum degradation rate temperature (T_max_) appears at 312 °C. The T_onset_ values decrease upon the incorporation of grape cane extract, and it is most pronounced in the case of TPS/WE15. In fact, DTG curve of TPS/WE15 displays a wide shoulder degradation peak, suggesting decomposition of bioactive components from extract, glycerol and TPS chains, all together. These data are in agreement with research work published by Agustin-Salazar et al. [34] and Ortiz-Vazquez et al. [43], where a decrease in thermal stability of PLA and butylated hydroxytoluene films with the addition of resveratrol as a bioactive component, respectively, was observed. Although the thermal stability of S films that contain the grape cane extracts is slightly reduced, T_onset_ is above 270 °C, which is significantly above the processing temperature region of TPS in the compression molding machine (140 °C), thus confirming that these formulations could be processed without risk of high thermal degradation of neat components.

### 2.5. Antioxidant Capacity

The grapevine cane extract used in the present study is mainly composed of the following stilbenoids: (*E*)-ε-vinifera and (*E*)-resveratrol. By another side, the solubilities of trans-resveratrol (stilbene model compound) in different alcohol solvents and water had been measured at different temperatures [44]. The authors found that its solubility increases with temperature but decreases along with carbon numbers in alcohol solvents, and the solubility in alcohol was higher than in water. That is why we selected water, pure methanol and its mixture with water as stilbene release media for antioxidant capacity measurements.

**Figure 5:**
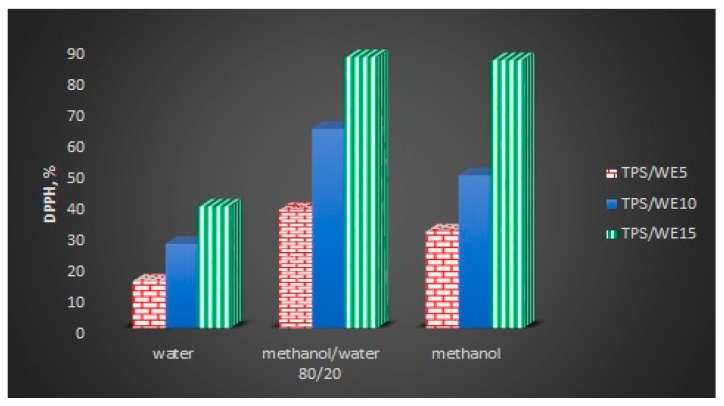
Antioxidant activity of TPS/WE samples.

The radical-scavenging activity of the neat TPS and TPS/WE samples was assessed by DPPH assay, using three extractive mediums for bioactive components from the starch matrix: water, methanol/water 80/20 *v*/*v* and methanol (Figure 5). Neat TPS does not show any antioxidant activity, whereas all TPS/WE samples exhibit the highest antioxidant activity when methanol/water 80/20 *v*/*v* is used as an extraction medium. This result is expected because grapevine cane extract is not soluble in water, whereas it has moderate solubility in pure alcohol and complete dissolution in alcohol/water 70/30 or 80/20 *v*/*v* mixture. The antioxidant capacity of TPS/WE samples increases with an increase of the extract content in materials. The radical-scavenger capacity ranged from 15 (TPS/WE5) to 39% (TPS/WE15) using water as a solvent, from 38 to 87 in methanol/water 80/20 *v*/*v*, and from 31 to 86% in absolute methanol, for 1000 µL of DPPH solution. High antioxidant capacity of obtained grape cane extract has been already proven in our previous paper [26] and supported in the literature by other authors [45,46], due to the presence of phenolic groups in extract itself. The IC_50_ (concentration required to scavenge 50% DPPH radicals) values of the oligostillbenes caraphenol A and α-viniferin A were determined and compared with Trolox antioxidant standard by Li et al. [47]. All compounds showed antioxidant activity in a dose-dependent manner, which agrees with results from this work. The authors reported that the antioxidant reaction could proceed by redox-mediated mechanisms (especially electron transfer and H+ -transfer) as well as non-redox-mediated mechanisms. In another study, the scavenging activity of 10 new stilbenoids isolated from the roots of *Caragana sinica* was measured. Only three of these compounds showed moderate DPPH scavenging activity and lipid peroxidation inhibitory activities with IC_50_ values ranging from 34.7 to 89.1 μM [48]. Regarding starch-based films, it was shown that the incorporation of orange peel oil/zein nanocapsules provided DPPH radical scavenging activity of 30% [49]. Yun at al. obtained 60% of DPPH scavenging activity when 4 wt% of Chinese bayberry was added into starch films [50]. On the other side, when a higher concentration of extract is included into the starch matrix (between 10 and 20%), high antioxidative activity can be obtained. For example, Pineros-Hernandez at al. obtained a similar antioxidant activity of starch/rosemary extract (20 wt% of rosemary extract) films to those in this work [51].

### 2.6. Microbiological Assay

**Figure 6:**
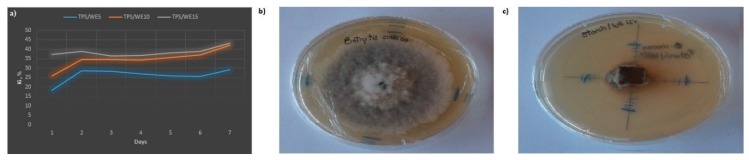
(**a**) Inhibition growth of *Botrytis cinerea* on S/WE samples; (**b**) Control sample—the growth of *Botrytis cinerea* after 7 days of incubation at 30 °C; (**c**) The growth of *Botrytis cinerea* in the presence of TPS/WE15 sample after 7 days of incubation at 30 °C.

The inhibition growth (IG) of the *Botrytis cinerea*, *Mucor indicus*, *Aspergillus niger*, *Rhizopus stolonifer,* and *Geotrichum candidum* on the control TPS, grape cane extract and TPS/WE samples was determined. TPS control sample does not show antifungal activity toward any of tested pathogens. On the other side, grape cane extract shows complete inhibition growth only of *Botrytis cinerea*. TPS/WE samples show moderate antifungal activity toward *Botrytis cinerea* by the reduction of the growth rate of fungi. The inhibition growth rate of *Botrytis cinerea* at the contact surface is in the range between 29% (TPS/W5) and 43% (TPS/WE15) (see Figure 6). As the concentration of extract in thermoplastic starch matrix is increasing, the inhibition growth of fungi is higher. Moreover, the spore germination has not been detected, which is important in the control of the phytopathogens, because a lack of spore germination inhibits reproduction and dissemination of fungi. This result implies that TPS/WE material can be used as a supportive layer in food packaging, but only directly placed in the contact zone with food products (fruits), and thus, prevents their further contamination or spoilage during storage and transport.

The antifungal activity of TPS/WE samples is mainly attributed to the presence of bioactive stilbenoids in the extract. In fact, high antifungal activity of resveratrol and moderate activity of viniferin toward *Botrytis cinerea* has already been proved by several authors [28,52,53]. On the other side, TPS/WE samples do not show any antifungal effect against other tested fungi pathogens. Regarding the antifungal activity of films containing resveratrol or viniferin, there is not enough data literature to be able to explain such selective antifungal behavior obtained in this work. Pastor et al. pointed out that chitosan-methylcellulose/resveratrol films did not have antifungal activity toward *Botrytis cinerea* and *Penicillium italicum*, due to the low release of resveratrol from the biopolymer matrix into the environment [54]. However, Lozano–Navarro et al. obtained moderate antifungal activity toward *Penicillum notatum*, *Aspergillus niger* and *Aspergillus fumigatus* [55].

**Figure 7:**
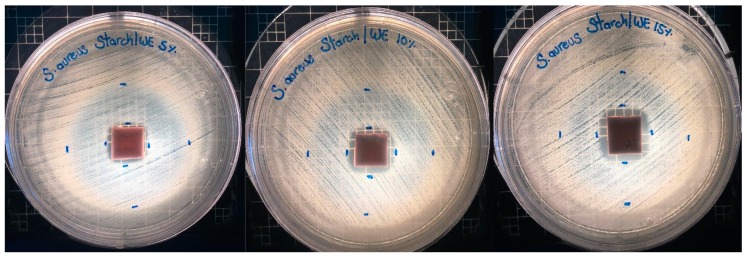
Antimicrobial activity of TPS/WE sample toward *S. aureus*.

Antimicrobial activity tests of TPS/WE samples toward *E. coli*, *S. aureus* and *Salmonella typhimurium* were also performed. The control TPS film does not show any antimicrobial activity, as it is expected. TPS/WE samples show neglected antimicrobial activity in the contact zone toward *E. coli* and moderate antimicrobial activity toward *S. aureus*. On the other side, samples do not show any antimicrobial activity toward *Salmonella typhimurium*. It is interesting to note that all samples tested against *S. aureus* give two halo zones, first related to 100% of growth inhibition in area of 13 × 13 mm (TPS/WE15) and moderate growth inhibition in area 35 × 35 mm (see Figure 7). These results are in agreement with the data in the literature. Li et al. pointed out that resveratrol was most efficient for the growth inhibition of *S. aureus* and less active toward *E.coli* and *C. albicans* [56]. Moreover, Paulo et al. observed higher antimicrobial activity of resveratrol toward Gram positive bacteria (*Bacillus* and *S. aureus*) than Gram negative bacteria (*E. coli*, *Salmonella* and *Klebsiella*), suggesting that the antimicrobial mechanism of resveratrol disrupts the microbial cell cycle, i.e., microbial growth, evidenced by changes in cell morphology and DNA contents [57].

## 3. Materials and Methods

### 3.1. Materials and Methods

Corn starch with a molecular weight of 50,000 g/mol was obtained from Corn Products Chile Inducorn S.A. Glycerol was purchased from OCN company (China).

### 3.2. Extraction Method from Grape cane Waste

The detailed extraction procedure and characterization of active components from grape vine (*Vitis vinifera* L.) canes were briefly described in a Chilean Patent [58]. Namely, Pinot Noir grape canes pruned in the winter of 2014 at De Neira Vineyard, Bio-Bio region, Chile, were used as a source of bioactive compounds. After storage for over 3 months at 19 °C ± 5 and 70% relative humidity, the grape canes were chopped in a Retsch grinder (model SM) at 300–2000 rpm and immersed in a reactor that contained ethanol/water solution (80:20 *v*/*v*) at 80 °C for 100 min. After solvent evaporation, the extract was collected and spray-dried using a BHS Büttner-Schilde-Haas AG dryer at a rate of 15 mL/min, operated with an inlet temperature of 160 ± 5 °C, outlet temperature at 60 ± 5 °C, and injected compressed air at 40 MPa. The spray-dried grape cane extract (WE) was stored under room temperature in aluminum containers. According to HPLC analysis, the main bioactive components of the obtained extract are trans-resveratrol (14.3 mg/L) and trans-ξ-viniferin (29.0 mg/L).

### 3.3. Preparation of Starch-Material

In order to obtain control thermoplastic starch, 500 g of corn starch was homogenized with 150 g of glycerol and 25 g of water at 45 °C and a speed rate of 2800 rpm in high-speed blade mixer (Cool Mixer, Labtech model LCM-24). Afterward, 25 g of homogenized starch was placed between two stainless steel plates that were covered with a teflon sheet. The dimension of the mold was 100 × 100 × 0.5 mm^3^. The starch samples were pressed in a Labtech LP-20B hydraulic press at an applied pressure of 70 bar for 3 min at 140 °C. The resulting material was cooled for 1 min before being unmolded. This material is coded as TPS. TPS/WE materials were prepared by homogenization of corn starch, water, glycerol, and WE at different concentrations (5, 10 and 15 wt% per mass of starch) in a high-speed blade mixer, following the same procedure as for control TPS. The concentration of glycerol and water was kept constant in all formulations. The code formulations were TPS/WE5, TPS/WE10 and TPS/WE15 for samples containing 5, 10 and 15 wt% of WE, respectively.

### 3.4. Characterization of Materials

#### 3.4.1. FTIR Analysis

FTIR spectra of thermoplastic starch-based materials were obtained at room temperature by Jasco FT/IR 400 spectrometer in the range of 4000–400 cm^−1^ at a resolution of 4 cm^−1^.

#### 3.4.2. SEM Analysis

The morphological analysis was performed by an ETEC autoscan SEM (Model U-1, University of Massachusetts; Worcester, MA). The samples were fixed in a sample holder and covered with a gold layer for 3 min using an Edwards S150 sputter coater (BOC Edwards, São Paulo, Brazil).

#### 3.4.3. Mechanical Analysis

The tensile test was performed by Instron dynamometer model 1185, equipped with a 1 kN load cell, according to the procedure described in ASTM D638 standard. The cross speed rate was 10 mm min^−1^. All measurements were carried out at room temperature and 50% of relative humidity. The reported data are the average values of six determinations. The obtained values of the tensile strength, elongation at break and Young’s modulus were within ± 10%.

#### 3.4.4. Thermal Analysis

The thermal stability of TPS and TPS/WE sheets was monitored by a NETZSCH TG 209 F3 Tarsus^®^ thermal analyzer. The measurements were carried out at a heating rate of 10 °C/min and under nitrogen atmosphere from ambient temperature to 500 °C. For each composition, the thermogravimetric tests were performed in duplicate.

#### 3.4.5. Antioxidant Capacity

For determination of the antioxidant capacity of biomaterials, the 2,2-diphenyl-1-picrylhydrazyl (DPPH) free radical was used, according to the methodology previously described by Ventura-Aguilar et al. [59]. Briefly, 1 cm^2^ of each TPS/WE material was macerated with three solvents: distilled water, methanol/water (80:20), and methanol, and centrifuged at 8500 rpm for 10 min. Twenty microliters of the supernatant were recovered and mixed with 750 µL of DPPH% (133.33 µL) and incubated for 30 min at room temperature. The absorbance was measured at 517 nm, using a Genesys 10s UV-VIS spectrophotometer. The results were expressed as a percentage of DPPH radical scavenging according to the following Equation (1), where Ab and As represents absorbance of the blank and sample, respectively.

% DPPH reduction = (Ab−As)/Ab × 100(1)

#### 3.4.6. In vitro Antimicrobial and Antifungal Activity

The antimicrobial assays were performed against 3 ATCC bacteria *E. coli* ATCC 25922, *Salmonella typhimurium* ATCC 14028 and *S. aureus* ATCC 25923. The inoculum was prepared using a direct colony suspension method in nutritive broth. The bacterial growth turbidity was established by the McFarland 0.5 method (1 x 108CFU/mL). A cotton swab was used to inoculate the nutritive agar, which was moistured with the bacterial suspension and distributed over the entire surface of the Petri plates. It was left to dry for 10 min, and afterward, the corresponding starch samples were placed. The inhibition zones were determined after 24 h of incubation at 35 ± 2 °C.

For antifungal assays, the microorganisms *Botrytis cinerea*, *Mucor indicus*, *Aspergillus niger*, *Rhizopus stolonifera,* and *Geotrichum candidum* were grown separately on Potato Dextrose Agar (PDA) Petri plates for a period of 3 weeks at 25 °C. Each starch sample (1cm × 1 cm) was placed on a PDA plate surface seeded with 5 mm of the fungal spore disc. The fungal plates were incubated at 25 °C for 7 days, and mycelial growth was measured daily using a Vernier caliper, in order to evaluate the diameter reached by the mycelium over time. Analyses were carried out in triplicate.

## 4. Conclusions

Grape cane extract obtained from viticulture residues due to its antifungal/antimicrobial properties was included in different ratios in thermoplastic starch materials by a compression molding technique. These materials were characterized by various techniques in order to evaluate their physical-chemical properties and potential usage in the food packaging sector. Materials containing the highest ratio of grape cane extract (15 wt%) showed sufficient thermal stability, moderate mechanical resistance and highest antifungal and antimicrobial activity, confirming that viticulture waste could be good source of natural, non-toxic active components in comparison to commonly used synthetic fungicides and could be efficiently incorporated into thermoplastic biopolymers, acting as a bioactive food packaging layer.

## Figures and Tables

**Table 1:** Thermogravimetric parameters obtained for TPS and TPS/WE samples.

Sample	W_L100_,%	W_L180_,%	T_onset_, °C	T_deg_, °C	Char Residue, °C
TPS	2	8	280	312	10
WE	3	14	220	240–390	36
TPS/WE5	2	9	273	312	14
TPS/WE10	2	9	271	314	17
TPS/WE15	2	9	269	319	19

## References

[1] PlasticsEurope Plastics-The facts 2017. https://www.plasticseurope.org/application/files/5715/1717/4180/Plastics_the_facts_2017_FINAL_for_website_one_page.pdf.

[2] Mexis S.F., Kontominas M.G., Batt C., Tortorello Lou M. (2014). PACKAGING | Active Food Packaging. Encyclopedia of Food Microbiology.

[3] Santagata G., Valerio F., Cimmino A., Dal Poggetto G., Masi M., Di Biase M., Malinconico M., Lavermicocca P., Evidente A. (2017). Chemico-physical and antifungal properties of poly(butylene succinate)/cavoxin blend: Study of a novel bioactive polymeric based system. Eur. Polym. J..

[4] Otoni C.G., Espitia P.J.P., Avena-Bustillos R.J., McHugh T.H. (2016). Trends in antimicrobial food packaging systems: Emitting sachets and absorbent pads. Food Res. Int..

[5] Garrido-Miranda K.A., Rivas B.L., Pérez -Rivera M.A., Sanfuentes E.A., Peña-Farfal C. (2018). Antioxidant and antifungal effects of eugenol incorporated in bionanocomposites of poly(3-hydroxybutyrate)-thermoplastic starch. LWT.

[6] Campos-Requena V.H., Rivas B.L., Pérez M.A., Figueroa C.R., Figueroa N.E., Sanfuentes E.A. (2017). Thermoplastic starch/clay nanocomposites loaded with essential oil constituents as packaging for strawberries − In vivo antimicrobial synergy over Botrytis cinerea. Postharvest Biol. Technol..

[7] Castillo L.A., Farenzena S., Pintos E., Rodríguez M.S., Villar M.A., García M.A., López O.V. (2017). Active films based on thermoplastic corn starch and chitosan oligomer for food packaging applications. Food Packag. Shelf Life.

[8] Mahieu A., Terrié C., Youssef B. (2015). Thermoplastic starch films and thermoplastic starch/polycaprolactone blends with oxygen-scavenging properties: Influence of water content. Ind. Crops Prod..

[9] Pola C.C., Medeiros E.A.A., Pereira O.L., Souza V.G.L., Otoni C.G., Camilloto G.P., Soares N.F.F. (2016). Cellulose acetate active films incorporated with oregano ( Origanum vulgare ) essential oil and organophilic montmorillonite clay control the growth of phytopathogenic fungi. Food Packag. Shelf Life.

[10] Wang H.J., Jo Y.H., An D.S., Rhim J.-W., Lee D.S. (2015). Properties of agar-based CO2 absorption film containing Na2CO3 as active compound. Food Packag. Shelf Life.

[11] Khan B., Bilal Khan Niazi M., Samin G., Jahan Z. (2017). Thermoplastic Starch: A Possible Biodegradable Food Packaging Material-A Review. J. Food Process. Eng..

[12] Zhang Y., Rempel C., Liu Q. (2014). Thermoplastic Starch Processing and Characteristics—A Review. Crit. Rev. Food Sci. Nutr..

[13] Heydari A., Alemzadeh I., Vossoughi M. (2013). Functional properties of biodegradable corn starch nanocomposites for food packaging applications. Mater. Des..

[14] Feng M., Yu L., Zhu P., Zhou X., Liu H., Yang Y., Zhou J., Gao C., Bao X., Chen P. (2018). Development and preparation of active starch films carrying tea polyphenol. Carbohydr. Polym..

[15] Vaezi K., Asadpour G., Sharifi H. (2019). Effect of ZnO nanoparticles on the mechanical, barrier and optical properties of thermoplastic cationic starch/montmorillonite biodegradable films. Int. J. Biol. Macromol..

[16] Jumaidin R., Sapuan S.M., Jawaid M., Ishak M.R., Sahari J. (2017). Effect of seaweed on mechanical, thermal, and biodegradation properties of thermoplastic sugar palm starch/agar composites. Int. J. Biol. Macromol..

[17] Kargarzadeh H., Johar N., Ahmad I. (2017). Starch biocomposite film reinforced by multiscale rice husk fiber. Compos. Sci. Technol..

[18] Chang P.R., Jian R., Yu J., Ma X. (2010). Fabrication and characterisation of chitosan nanoparticles/plasticised-starch composites. Food Chem..

[19] Salaberria A.M., Diaz R.H., Labidi J., Fernandes S.C.M. (2015). Role of chitin nanocrystals and nanofibers on physical, mechanical and functional properties in thermoplastic starch films. Food Hydrocoll..

[20] Fabra M.J., López-Rubio A., Ambrosio-Martín J., Lagaron J.M. (2016). Improving the barrier properties of thermoplastic corn starch-based films containing bacterial cellulose nanowhiskers by means of PHA electrospun coatings of interest in food packaging. Food Hydrocoll..

[21] Ali A., Chen Y., Liu H., Yu L., Baloch Z., Khalid S., Zhu J., Chen L. (2019). Starch-based antimicrobial films functionalized by pomegranate peel. Int. J. Biol. Macromol..

[22] Oleyaei S.A., Almasi H., Ghanbarzadeh B., Moayedi A.A. (2016). Synergistic reinforcing effect of TiO 2 and montmorillonite on potato starch nanocomposite films: Thermal, mechanical and barrier properties. Carbohydr. Polym..

[23] Ghanbari A., Tabarsa T., Ashori A., Shakeri A., Mashkour M. (2018). Preparation and characterization of thermoplastic starch and cellulose nanofibers as green nanocomposites: Extrusion processing. Int. J. Biol. Macromol..

[24] Ostafińska A., Mikešová J., Krejčíková S., Nevoralová M., Šturcová A., Zhigunov A., Michálková D., Šlouf M. (2017). Thermoplastic starch composites with TiO 2 particles: Preparation, morphology, rheology and mechanical properties. Int. J. Biol. Macromol..

[25] Vergara C., von Baer D., Mardones C., Wilkens A., Wernekinck K., Damm A., Macke S., Gorena T., Winterhalter P. (2012). Stilbene Levels in Grape cane of Different Cultivars in Southern Chile: Determination by HPLC-DAD-MS/MS Method. J. Agric. Food Chem..

[26] Sáez V., Pastene E., Vergara C., Mardones C., Hermosín-Gutiérrez I., Gómez-Alonso S., Gómez M.V., Theoduloz C., Riquelme S., von Baer D. (2018). Oligostilbenoids in Vitis vinifera L. Pinot Noir grape cane extract: Isolation, characterization, in vitro antioxidant capacity and anti-proliferative effect on cancer cells. Food Chem..

[27] Gorena T., Mardones C., Vergara C., Saez V., von Baer D., Ebeler S.B., Sacks G., Vidal S., Winterhalter P. (2015). Evaluation of the Potential of Grape canes as a Source of Bioactive Stilbenoids. Advances in Wine Research.

[28] Schnee S., Queiroz E.F., Voinesco F., Marcourt L., Dubuis P.-H., Wolfender J.-L., Gindro K. (2013). Vitis vinifera Canes, a New Source of Antifungal Compounds against Plasmopara viticola, Erysiphe necator, and Botrytis cinerea. J. Agric. Food Chem..

[29] Corrales M., Han J.H., Tauscher B. (2009). Antimicrobial properties of grape seed extracts and their effectiveness after incorporation into pea starch films. Int. J. Food Sci. Technol..

[30] Xu Y., Scales A., Jordan K., Kim C., Sismour E. (2017). Starch nanocomposite films incorporating grape pomace extract and cellulose nanocrystal. J. Appl. Polym. Sci..

[31] Gutiérrez T.J., Herniou-Julien C., Álvarez K., Alvarez V.A. (2018). Structural properties and in vitro digestibility of edible and pH-sensitive films made from guinea arrowroot starch and wastes from wine manufacture. Carbohydr. Polym..

[32] Castillo L.A., López O.V., García M.A., Barbosa S.E., Villar M.A. (2019). Crystalline morphology of thermoplastic starch/talc nanocomposites induced by thermal processing. Heliyon.

[33] Musa M., Yoo M., Kang T., Kolawole E., Ishiaku U., Yakubu M., Whang D. (2013). Characterization and Thermomechanical Properties of Thermoplastic Potato Starch. Res. Rev. J. Eng. Technol..

[34] Agustin-Salazar S., Gamez-Meza N., Medina-Juàrez L.À., Soto-Valdez H., Cerruti P. (2014). From Nutraceutics to Materials: Effect of Resveratrol on the Stability of Polylactide. ACS Sustain. Chem. Eng..

[35] Billes F., Mohammed-Ziegler I., Mikosch H., Tyihák E. (2007). Vibrational spectroscopy of resveratrol. Spectrochim. Acta Part. A Mol. Biomol. Spectrosc..

[36] Zhu F. (2015). Interactions between starch and phenolic compound. Trends Food Sci. Technol..

[37] Davoodi M., Kavoosi G., Shakeri R. (2017). Preparation and characterization of potato starch-thymol dispersion and film as potential antioxidant and antibacterial materials. Int. J. Biol. Macromol..

[38] Nogueira G.F., Soares C.T., Cavasini R., Fakhouri F.M., de Oliveira R.A. (2019). Bioactive films of arrowroot starch and blackberry pulp: Physical, mechanical and barrier properties and stability to pH and sterilization. Food Chem..

[39] Homayouni H., Kavoosi G., Nassiri S.M. (2017). Physicochemical, antioxidant and antibacterial properties of dispersion made from tapioca and gelatinized tapioca starch incorporated with carvacrol. LWT.

[40] Silva Â., Duarte A., Sousa S., Ramos A., Domingues F.C. (2016). Characterization and antimicrobial activity of cellulose derivatives films incorporated with a resveratrol inclusion complex. LWT.

[41] da Silva R.D., Teixeira J.A., Nunes W.D.G., Zangaro G.A.C., Pivatto M., Caires F.J., Ionashiro M. (2017). Resveratrol: A thermoanalytical study. Food Chem..

[42] Öğünç Y., Demirel M., Yakar A., İncesu Z. (2017). Vincristine and ε-viniferine-loaded PLGA-b-PEG nanoparticles: Pharmaceutical characteristics, cellular uptake and cytotoxicity. J. Microencapsul..

[43] Ortiz-Vazquez H., Shin J., Soto-Valdez H., Auras R. (2011). Release of butylated hydroxytoluene (BHT) from Poly(lactic acid) films. Polym. Test..

[44] Sun X., Peng B., Yan W. (2008). Measurement and correlation of solubility of trans-resveratrol in 11 solvents at T= (278.2, 288.2, 298.2, 308.2, and 318.2) K. J. Chem. Thermodyn..

[45] Ju Y., Zhang A., Fang Y., Liu M., Zhao X., Wang H., Zhang Z. (2016). Phenolic compounds and antioxidant activities of grape canes extracts from vineyards. Spanish, J. Agric. Res..

[46] Zhang A., Fang Y., Wang H., Li H., Zhang Z. (2011). Free-Radical Scavenging Properties and Reducing Power of Grape cane Extracts from 11 Selected Grape Cultivars Widely Grown in China. Molecules.

[47] Li X., Xie Y., Xie H., Yang J., Chen D. (2018). π-π Conjugation Enhances Oligostilbene’s Antioxidant Capacity: Evidence from α-Viniferin and Caraphenol, A. Molecules.

[48] Jin Q., Han X.H., Hong S.S., Lee C., Choe S., Lee D., Kim Y., Hong J.T., Lee M.K., Hwang B.Y. (2012). Antioxidative oligostilbenes from Caragana sinica. Bioorg. Med. Chem. Lett..

[49] Wang Y., Zhang R., Ahmed S., Qin W., Liu Y. (2019). Preparation and Characterization of Corn Starch Bio-Active Edible Packaging Films Based on Zein Incorporated with Orange-Peel Oil. Antioxidants.

[50] Yun D., Cai H., Liu Y., Xiao L., Song J., Liu J. (2019). Development of active and intelligent films based on cassava starch and Chinese bayberry ( Myrica rubra Sieb. et Zucc.) anthocyanins. RSC Adv..

[51] Piñeros-Hernandez D., Medina-Jaramillo C., López-Córdoba A., Goyanes S. (2017). Edible cassava starch films carrying rosemary antioxidant extracts for potential use as active food packaging. Food Hydrocoll..

[52] Langcake P., Pryce R.J. (1976). The production of resveratrol by Vitis vinifera and other members of the Vitaceae as a response to infection or injury. Physiol. Plant. Pathol..

[53] Adrian M., Jeandet P. (2012). Effects of resveratrol on the ultrastructure of Botrytis cinerea conidia and biological significance in plant/pathogen interactions. Fitoterapia.

[54] Pastor C., Sánchez-González L., Chiralt A., Cháfer M., González-Martínez C. (2013). Physical and antioxidant properties of chitosan and methylcellulose based films containing resveratrol. Food Hydrocoll..

[55] Lozano-Navarro J., Díaz-Zavala N., Velasco-Santos C., Martínez-Hernández A., Tijerina-Ramos B., García-Hernández M., Rivera-Armenta J., Páramo-García U., Reyes-de la Torre A. (2017). Antimicrobial, Optical and Mechanical Properties of Chitosan–Starch Films with Natural Extracts. Int. J. Mol. Sci..

[56] Li X.-Z., Wei X., Zhang C.-J., Jin X.-L., Tang J.-J., Fan G.-J., Zhou B. (2012). Hypohalous acid-mediated halogenation of resveratrol and its role in antioxidant and antimicrobial activities. Food Chem..

[57] Paulo L., Ferreira S., Gallardo E., Queiroz J.A., Domingues F. (2010). Antimicrobial activity and effects of resveratrol on human pathogenic bacteria. World, J. Microbiol. Biotechnol..

[58] Mardones C., Von Baer D., Vergara C., Fuentealba C., Escobar D., Riquelme S. (2014). Un procedimiento para aumentar el contenido de estilbenos, esencialmente resveratrol, en sarmientos provenientes de las podas de Vitis vinífera. Chilean Patent.

[59] Ventura-Aguilar R.I., Bautista-Baños S., Flores-García G., Zavaleta-Avejar L. (2018). Impact of chitosan based edible coatings functionalized with natural compounds on Colletotrichum fragariae development and the quality of strawberries. Food Chem..

